# Procalcitonin in Acute Pancreatitis: Diagnostic Utility for Differentiating Infected from Sterile Pancreatic Necrosis

**DOI:** 10.3390/life16101753

**Published:** 2026-10-19

**Authors:** Geetha Kandasamy, Lavanya Selvaraj, Yashvi Ambrish Pathak, Sangavi Anandan, Arun Prasannan, Rehab Ahmed, Vasudevan Mani

**Affiliations:** 1 Department of Clinical Pharmacy, College of Pharmacy, King Khalid University, Abha 61421, Saudi Arabia; 2 Department of Pharmacy Practice, PSG College of Pharmacy, Peelamedu, Coimbatore 641004, Tamil Nadu, India; 3 Department of Gastroenterology, GKNM Hospital, Nethaji Road, Pappanaickenpalayam, Coimbatore 641037, Tamil Nadu, India; 4 Department of Natural Products and Alternative Medicine, Faculty of Pharmacy, University of Tabuk, Tabuk 71491, Saudi Arabia; 5 Department of Pharmacology and Toxicology, College of Pharmacy, Qassim University, Buraydah 51452, Saudi Arabia

**Keywords:** acute pancreatitis, procalcitonin, infection, biomarkers, pancreatitis, antimicrobial stewardship

## Abstract

Acute pancreatitis (AP) is an acute inflammatory disorder of the pancreas that disrupts its normal function and often requires hospitalization because of severe abdominal pain. While most patients recover without major complications, some develop pancreatic necrosis, infected pancreatic necrosis (IPN), persistent organ failure, and other severe complications associated with increased morbidity and mortality. A key clinical challenge is distinguishing infected pancreatic necrosis from sterile pancreatic inflammation, as delayed or inaccurate diagnosis may result in inappropriate antibiotic use and poorer clinical outcomes. This review aimed to evaluate the diagnostic and prognostic utility of procalcitonin (PCT) in differentiating infected pancreatic necrosis from sterile inflammation and to assess its role in supporting antimicrobial decision-making in patients with AP. A structured narrative literature review was conducted using PubMed, Embase, Scopus, and Web of Science to identify English-language human studies, meta-analyses, randomized controlled trials, and international guidelines published between January 2000 and December 2025. A total of 60 studies were included in the narrative synthesis. The reviewed evidence indicates that PCT may provide greater diagnostic specificity for bacterial infection than conventional inflammatory markers such as C-reactive protein (CRP) and leukocyte count in selected clinical settings. Elevated or persistently increasing PCT concentrations have been associated with IPN, sepsis, organ failure, and increased mortality. Furthermore, PCT-guided antibiotic stewardship strategies may reduce unnecessary antibiotic exposure without adversely affecting clinical outcomes in selected settings. Overall, PCT represents a potentially valuable adjunctive biomarker for differentiating IPN from sterile inflammation in patients with AP. When interpreted alongside clinical assessment and imaging findings, PCT may facilitate the assessment of infection risk, prognostic stratification, and antimicrobial stewardship. However, further large-scale prospective multicenter studies are warranted to establish standardized diagnostic thresholds and optimize its integration into routine clinical practice.

## 1. Introduction

AP is a major cause of gastrointestinal hospital admissions worldwide [1,2], with an estimated incidence of 34 cases per 100,000 persons annually [3]. While most cases are mild, approximately 20% progress to severe acute pancreatitis (SAP) with necrosis, systemic complications, and persistent organ failure [4,5]. The change to IPN is a major turning point. Sterile necrosis carries a baseline mortality rate of 5–10%; however, superinfection of necrotic tissue drives mortality to between 20% and 40% [6,7]. When IPN coincides with persistent organ failure-designated as “critical acute pancreatitis”—mortality can exceed 40%, underscoring the paramount importance of prompt infection identification [8,9]. Yet, differentiating the sterile systemic inflammatory response syndrome (SIRS) that dominates the early phase of SAP from secondary bacterial sepsis poses a formidable clinical challenge [10]. Standard clinical parameters such as fever, tachycardia, tachypnea and leukocytosis lack specificity in SAP, as sterile inflammation can closely mimic bacterial sepsis [11]. Although CRP is widely used for severity assessment, its delayed peak at 48–72 h limits its usefulness for early diagnosis [11,12]. Although extraluminal gas on contrast-enhanced computed tomography (CT) is pathognomonic for infection, it appears in only a minority of IPN cases, rendering standard imaging largely insensitive for early detection [1]. CT-guided fine-needle aspiration (FNA) is invasive, carries a risk of false-negative results or iatrogenic infection, and has consequently been removed from routine recommendations in modern guidelines [1,13]. Amidst these diagnostic challenges, PCT has emerged as a potential adjunctive biomarker for assessing infectious complications in AP. Being an inactive propeptide of calcitonin, PCT is rapidly released in response to bacterial endotoxins and proinflammatory cytokines; its levels generally begin to increase within 2–4 h of a bacterial stimulus [11]. This relatively rapid kinetic pattern has prompted investigation of PCT as an adjunctive biomarker for differentiating sterile inflammation from infected necrosis, although its diagnostic performance relative to other biomarkers varies across clinical settings [14]. While previous systematic reviews have primarily evaluated the pooled diagnostic accuracy and prognostic associations of PCT in AP [11], the present narrative review integrates evidence on PCT kinetics, clinically relevant thresholds, comparative findings with other biomarkers, and its potential application in antimicrobial stewardship. Specifically, the review synthesizes evidence on clinically relevant PCT thresholds and kinetics and interprets these findings in the context of contemporary recommendations, including the 2025 revised IAP (International Association of Pancreatology) guidelines [15]. In addition, it examines factors that may influence PCT interpretation, such as renal dysfunction, and discusses how PCT may support antimicrobial stewardship and contemporary clinical decision-making in patients with suspected IPN [15,16]. Accordingly, this review evaluates the diagnostic and prognostic utility of PCT in AP, with particular emphasis on its potential role in differentiating infected from sterile pancreatic necrosis, interpreting PCT thresholds and kinetics, and supporting antibiotic decision-making and antimicrobial stewardship.

## 2. Methodology

### 2.1. Review Design and Literature Search

This study was designed as a structured narrative review to synthesise the available evidence on the role of PCT in AP, with particular emphasis on differentiating IPN from sterile pancreatic inflammation and on the potential role of PCT in antimicrobial stewardship. The structured narrative approach was selected to accommodate the heterogeneity of the available evidence, including diagnostic studies, observational studies, randomized controlled trials (RCT), evidence syntheses, and clinical practice guidelines addressing different clinical applications of PCT.

To enhance the transparency and comprehensiveness of the narrative review, a structured literature search was conducted in PubMed, Embase, Scopus, and Web of Science for articles published between January 2000 and May 2026. This period was selected to capture the evolution of evidence regarding PCT as a biomarker of infection, inflammation, disease severity, and antimicrobial management in AP.

Search terms included combinations of “procalcitonin”, “PCT”, “acute pancreatitis”, “infected pancreatic necrosis”, “infected necrosis”, “sterile necrosis”, “diagnosis”, “prognosis”, “biomarkers”, “antimicrobial stewardship”, and “antibiotic therapy”, combined using standard Boolean operators. A representative PubMed search strategy was: (“Procalcitonin” OR “PCT”) AND (“Acute Pancreatitis” OR “Acute Pancreatitis, Necrotizing”) AND (“Infected Pancreatic Necrosis” OR “Infected Necrosis” OR “Sterile Necrosis” OR “Pancreatic Necrosis” OR “Infection” OR “Prognosis” OR “Severity” OR “Biomarkers”). For PubMed, Medical Subject Headings (MeSH), where applicable, and free-text terms were used. Equivalent search strategies were adapted for Embase, Scopus, and Web of Science according to their respective indexing conventions. Reference lists of included studies and relevant review articles were also manually screened to identify additional eligible publications.

### 2.2. Eligibility Criteria

Evidence was considered eligible when it was relevant to the clinical role of PCT in adult patients with AP, including evidence from RCT, prospective and retrospective observational studies, diagnostic studies, evidence syntheses, and international clinical practice guidelines. Relevant outcomes included diagnosis of IPN, disease severity, organ dysfunction, mortality, infection, comparison with other biomarkers, and antimicrobial use. Studies were excluded if they were non-English publications, editorials or opinion pieces without relevant original data, conference abstracts, non-peer-reviewed material, grey literature, single-case reports, animal or preclinical studies, pharmacokinetic/pharmacodynamic-only studies, or studies that did not report a PCT-related diagnostic, prognostic, or antimicrobial-stewardship outcome relevant to AP.

### 2.3. Literature Screening and Data Extraction

**Figure 1:**
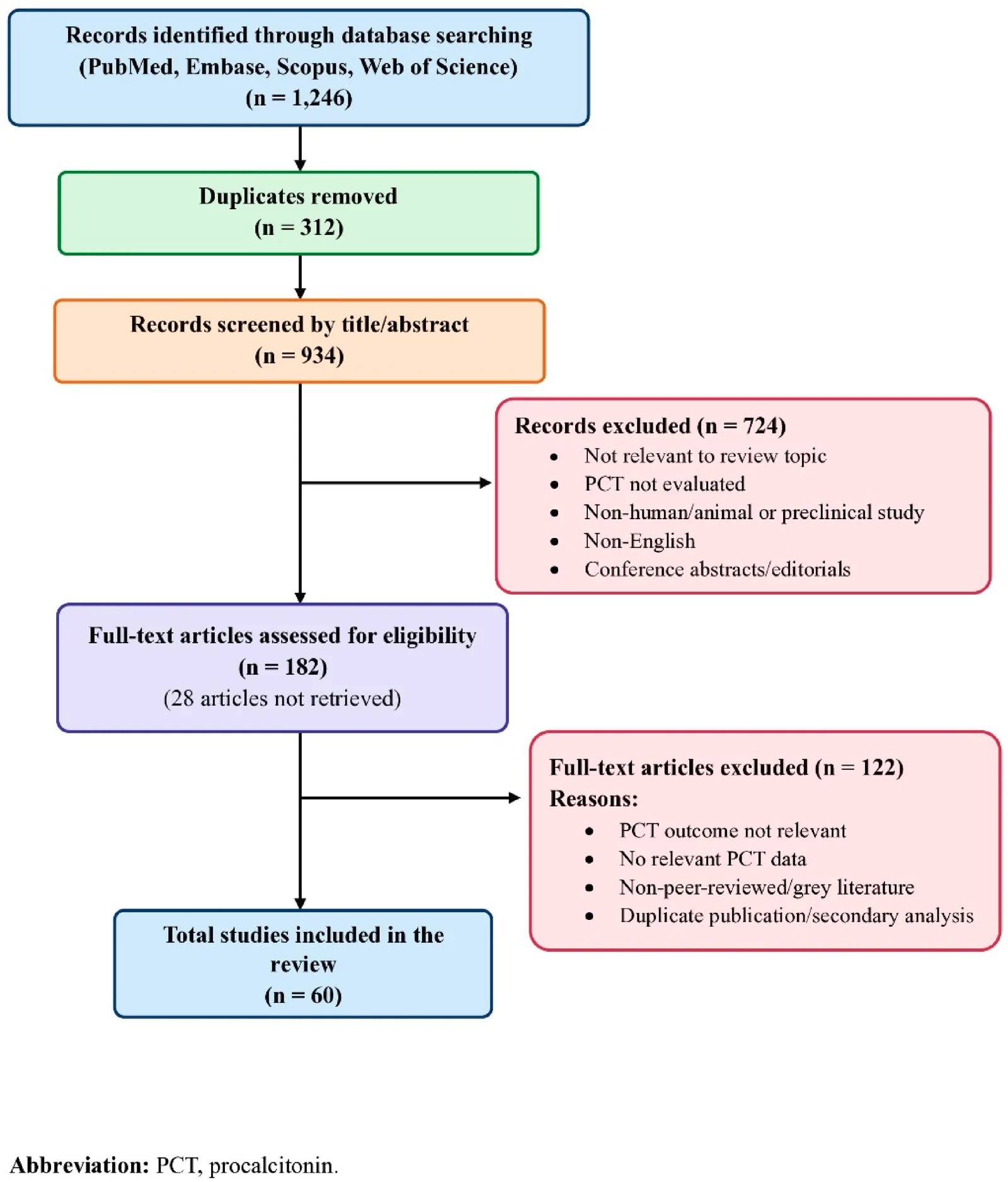
Flow diagram of the literature identification, screening, and study selection process for the structured narrative review.

The identified literature was screened for relevance to the review objectives. Two reviewers independently assessed the titles and abstracts of potentially relevant publications and subsequently evaluated the corresponding full texts for inclusion. Disagreements were resolved through discussion and consensus. A total of 1246 records were identified, of which 312 duplicates were removed. Of the remaining 934 records, 724 were excluded during initial screening. Full-text retrieval was sought for 210 publications; 28 could not be retrieved, leaving 182 publications for full-text assessment. Following full-text evaluation, 122 publications were excluded, resulting in 60 publications being included in the narrative synthesis Figure 1.

For each included publication, information was extracted on study design, clinical setting, patient population, sample size, PCT assay method and threshold, comparator biomarker(s), timing and frequency of PCT measurement, relevant clinical outcomes, follow-up duration, and principal findings. Methodological characteristics relevant to interpretation were also recorded when reported, including prospective versus retrospective design, single versus serial PCT measurement, blinding, independent outcome adjudication, outcome/reference-standard definitions, and relevant features of clinical-care standardization or antibiotic protocols. When these characteristics were not reported in the original publication, they were recorded as “Not reported.”

### 2.4. Narrative Synthesis

The included evidence was synthesized narratively and thematically according to the principal clinical applications of PCT: diagnosis and early prediction of IPN, prognostic assessment, and PCT-guided antimicrobial stewardship. Differences in study design, patient population, PCT measurement strategies, thresholds, reference standards, and treatment protocols were considered when interpreting the findings. No quantitative pooling or meta-analysis was performed.

## 3. Results

### 3.1. Pathophysiological Basis of Infection and Pct Elevation in Acute Pancreatitis

AP pathogenesis proceeds through a complex cascade, premature trypsin activation initiates pancreatic autodigestion, leading to release of damage-associated molecular patterns (DAMPs) that amplify immune signaling beyond local injury [3,14]. These signals trigger systemic inflammatory responses mediated by Interleukin- 1beta (IL-1*β*), Interleukin-6 (IL-6), Interleukin-8 (IL-8), and Tumor Necrosis Factor- alpha (TNF-*α*), leading to sterile inflammation [17,18]. Consequently, early clinical features resemble sepsis, explaining why clinical parameters fail to differentiate sterile SIRS from infection [11,19]. The intensity of this inflammatory response determines early organ dysfunction risk [17,20]. Importantly, sterile inflammation may produce some PCT elevation, but levels are generally lower than those observed with bacterial infection [11].

**Figure 2:**
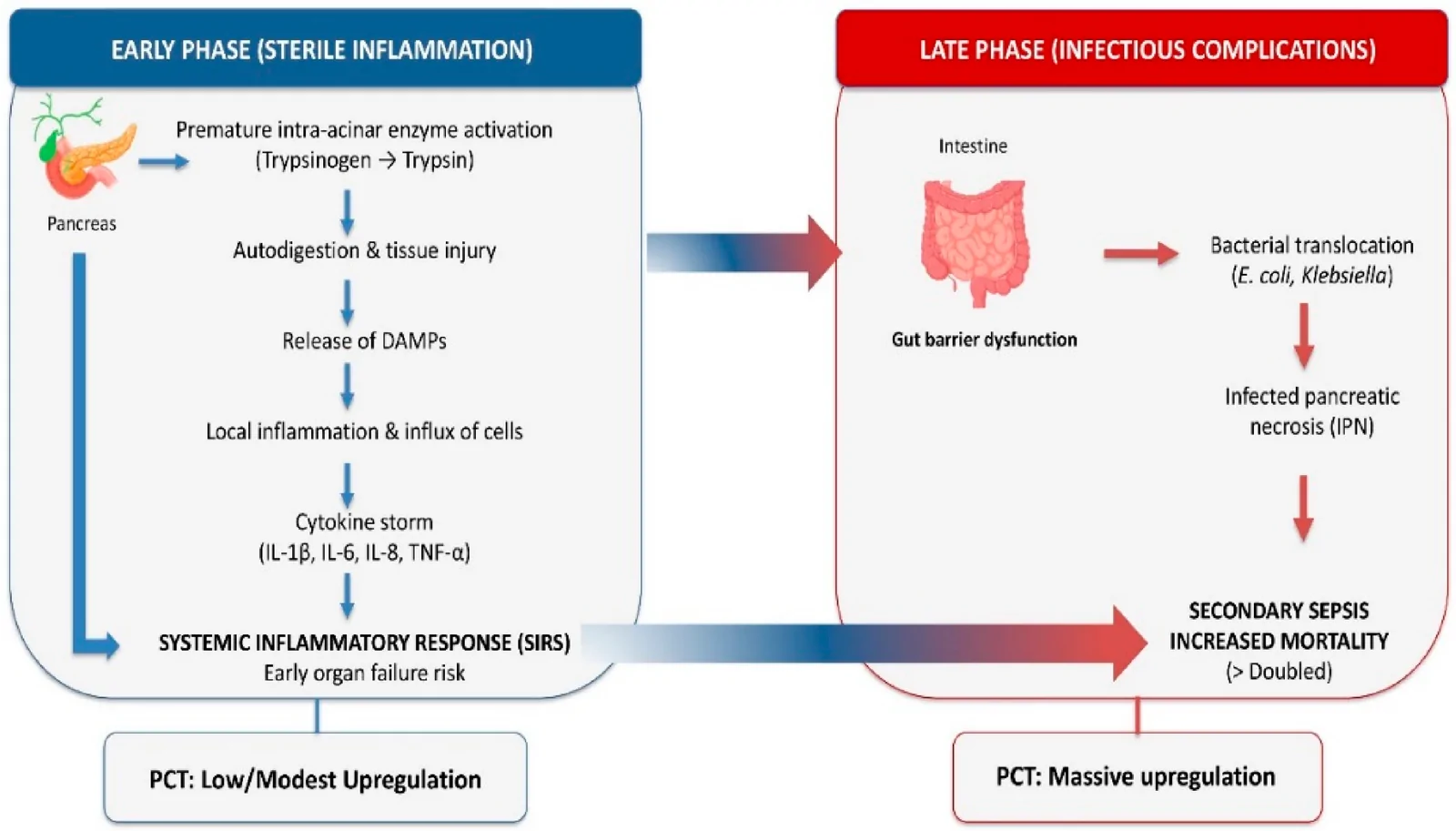
Pathophysiology progression of AP from sterile inflammation to infectious complications and its association with PCT dynamics. Abbreviations: DAMPs, damage-associated molecular patterns; SIRS, systemic inflammatory response syndrome; IPN, infected pancreatic necrosis; TNF-α, tumor necrosis factor-alpha; IL-1β, interleukin-1 beta; IL-6, interleukin-6; IL-8, Interleukin-8; PCT, procalcitonin; early phase is characterized by sterile inflammation and cytokine-mediated SIRS with low to modest PCT elevation, whereas late phase involves bacterial translocation, IPN, and secondary sepsis with marked PCT upregulation.

During the late phase, gut barrier failure results primarily from hypovolemia and splanchnic hypoperfusion, increasing intestinal permeability and enabling bacterial translocation into necrotic pancreatic tissue [13,14,18]. Infection of previously sterile necrosis leads to IPN and secondary sepsis, which worsens prognosis, with mortality more than doubling and rising from 22% with organ failure alone to 43% when infection coexists with organ failure [7,9,21]. Thus, the critical clinical transition is the shift from sterile inflammation to infection, where rising PCT levels may provide useful information to support antimicrobial decision-making [11]. The proposed progression from early sterile inflammation to late infectious complications, together with the corresponding changes in PCT expression, is illustrated in Figure 2.

### 3.2. Biology and Kinetics of Procalcitonin

PCT is a highly specific 116-amino acid glycoprotein serving as the precursor propeptide for calcitonin [22]. Under healthy physiological conditions, PCT is synthesized mostly by thyroid C-cells and undergoes rapid intracellular proteolytic cleavage. Consequently, circulating prohormone levels remain negligible, below the standard detection limit of 0.05–0.1 ng/mL [23,24].

During states of severe systemic bacterial inflammation, PCT biological production alters dramatically. Through “hormokine” expression, PCT synthesis becomes ubiquitous across extrathyroidal parenchymal tissues, including the liver, kidneys, adipose tissue, and skeletal muscle [6,22]. This constitutive synthesis bypasses standard intracellular cleavage, resulting in massive release of intact PCT directly into the systemic circulation [25].

Extrathyroidal PCT gene expression is primarily induced by bacterial endotoxins (e.g., lipopolysaccharide) and specific proinflammatory cytokines, notably TNF-*α*, IL-1*β*, and IL-6 [24]. A critical differentiating feature of PCT biology is its divergent regulation. While proinflammatory cytokines strongly stimulate PCT release, interferon-gamma (IFN-*γ*), secreted extensively during viral infections, actively attenuates and suppresses PCT production [24,26]. This biological regulation may contribute to the stronger association of PCT with bacterial infection than with some viral conditions [25,27].

PCT’s kinetic profile is exceptionally suited for acute diagnostics. Serum PCT levels rise measurably within 2–4 h following substantial bacterial stimulus, appearing significantly earlier than CRP [11,24]. Circulating PCT levels typically peak between 24 and 48 h [23,24]. With an estimated half-life of 25–30 h, PCT concentrations decline rapidly once the infectious stimulus is controlled [26,27]. Although sterile necrosis can also produce substantial PCT elevations through systemic inflammatory activation, available evidence suggests that concentrations may be higher in bacterial infection than in isolated sterile inflammation in some clinical settings [4,6].

### 3.3. Diagnostic Challenges in Acute Pancreatitis

AP clinical management depends on accurate differentiation between sterile inflammation and IPN. Standard clinical signs of infection (fever, tachycardia, tachypnea) possess high sensitivity but poor diagnostic specificity in AP [14]. The massive sterile inflammation associated with SAP induces SIRS, clinically indistinguishable from true bacterial sepsis [23,28].

While CRP remains the traditional laboratory marker, its diagnostic accuracy is hampered by latency. CRP levels ≥ 150 mg/L indicate severe disease but fail to distinguish between sterile and infected necrosis within the first 48 h of admission [2,14]. Leukocytosis is similarly non-specific, serving as an expected component of the sterile SIRS response [19]. Comprehensive scoring systems like Acute Physiology and Chronic Health Evaluation II (APACHE II) and Ranson’s criteria require 48 h to complete and do not specifically predict bacterial infection [4,24].

Contrast-enhanced computed tomography (CECT) remains the anatomical gold standard for assessing pancreatic necrosis but has limitations in early detection. Because necrosis may not be apparent until 72–96 h after symptom onset, early imaging can underestimate disease severity [24]. Macroscopic CT findings for sterile and infected necrosis are often identical. The truly pathognomonic “air bubble” sign (extraluminal gas) is present in only 20–50% of documented IPN cases [19,24]. Repeated CT scanning exposes vulnerable patients to significant radiation burdens and contrast-induced nephrotoxicity risks [14,24].

Failure to distinguish sterile from infected necrosis leads to errors like the unnecessary use of empiric broad-spectrum antibiotics in patients with sterile inflammation. Observational studies report antibiotic use in up to 63% of AP cases, often inappropriately, contributing to antimicrobial resistance, increased healthcare costs, and complications such as *Clostridioides difficile* infection [6,28,29]. Prophylactic antibiotics have not been shown to reduce IPN incidence or mortality and are discouraged by modern guidelines [1,14]. Conversely, failure to definitively diagnose IPN promptly can be fatal. Infected necrosis associates with a mortality rate of 20–40%, compared to <10% for sterile necrosis [24,30]. Mortality escalates if interventions are delayed in uncontrolled sepsis [28]. These challenges highlight the clinical need for biomarkers that can provide additional information for distinguishing bacterial infection from sterile inflammation in selected patients with AP.

### 3.4. Clinical Evidence Supporting Pct

PCT has been evaluated in numerous prospective studies and meta-analyses of AP over the past two decades. Although PCT has been associated with overall disease severity, its main clinical utility is to provide adjunctive information for differentiating sterile from IPN and for supporting early assessment of organ failure [14,31,32].

During the initial stage of AP (first 24–48 h), available evidence suggests that PCT may provide earlier risk-stratification information than conventional markers such as CRP in selected clinical settings, consistent with its relatively rapid kinetic profile [14,33]. Prospective studies and systematic reviews have reported associations between PCT concentrations and disease severity, with discriminatory value for differentiating sterile from infected necrosis and for identifying patients at increased risk of severe complications [14,31,32]. However, the magnitude of the diagnostic performance of PCT varies across studies according to patient population, timing of measurement, disease severity, diagnostic threshold, and reference standard [11,31,32]. In patients with mild or uncomplicated AP, the discriminatory value of individual inflammatory biomarkers may be less pronounced; IL-6, for example, has demonstrated useful early information for severity stratification in some studies [34].

The diagnostic performance of PCT for IPN reported in selected prospective studies and systematic reviews is summarized in Table 1. The available evidence includes both individual cohort studies and pooled analyses, but differences in study design, timing of sampling, reference standards, and thresholds limit direct comparison across studies.

PCT may also provide adjunctive information in the evaluation of suspected IPN, as higher or persistently elevated concentrations have been reported in some studies of infected compared with sterile necrosis [14,29,31,33]. Serial PCT measurements may provide additional prognostic information, with persistent elevation or an insufficient decline being associated with poorer outcomes, whereas decreasing concentrations have been associated with clinical improvement and treatment response [14,35].

Selected studies evaluating the prognostic performance of PCT for disease severity, organ failure, mortality, or composite infectious complications are summarized in Table 2. These studies illustrate the variability in PCT thresholds, sampling schedules, and clinical endpoints used to assess prognostic utility. Optimal PCT thresholds vary according to the study population, timing of assessment, assay, and clinical endpoint. Lower thresholds, including approximately 0.5 ng/mL, have been evaluated for infection-risk assessment and antibiotic-related decision-making, whereas higher thresholds have been investigated for predicting severe complications and infected necrosis [14,30,36,37]. These differences indicate that a single universal PCT cut-off cannot be applied across all clinical settings. Current evidence therefore supports interpretation of PCT in conjunction with clinical findings and other investigations, with serial trends potentially providing more useful information than reliance on a single measurement [27,35].

**Table 1:** Diagnostic and early-prediction accuracy of PCT for infected pancreatic necrosis.

Author	Year	Design and Setting	n (Events)	PCT Sampling (Single vs. Serial)	Cut-Off (ng/mL)	Sens (%)	Spec (%)	Outcome Definition/Reference Standard	Blinding	Independent Adjudication	Principal Limitations
Rau et al. [14]	2007	Prospective, international multicentre (5 European surgical centres); predicted SAP with SIRS, enrolled ≤ 96 h from symptom onset	104 IPN = 17 (16%)	Serial: daily for up to 21 days, then weekly; semi-automated chemiluminescence immunoassay (LUMITEST-PCT), run in real time at each centre Analysed as peak over ≥ 2 consecutive days (overall) and as peak on day 3 or 4 (early)	Overall ≥ 4.0 * Day 3–4 ≥ 1.5 *	65 82	89 69	Bacteriologically proven pancreatic infection (guided FNA and/or intra-operative findings) AUC 0.78 (overall)	Not reported. PCT run in real time.	Not reported. CRFs were source-checked at 6-monthly monitoring visits (data monitoring, not outcome adjudication)	Only 17 IPN events. Antibiotics given to 98% of patients. Data-derived cut-offs. Centre-specific PCT differences. BRAHMS support (author consultancy; monitoring visits)
Chen et al. [32]	2017	Retrospective single-centre cohort (Harbin, China); consecutive necrotising AP (moderately severe and severe), 2012–2016; 3-month follow-up	215 IPN = 87 (40.5%)	Single early window: maximum PCT value within the first 48 h of admission (not followed serially)	1.39 *	60.9	75.0	IPN arising a median 13.5 days after admission; positive culture (FNA or intervention) and/or CT gas AUC 0.674 (95% CI 0.596–0.752); combined PCT + CRP + HCT + BUN AUC 0.789 (sens 67.8, spec 77.3)	Not reported. (retrospective database analysis)	Not reported.	Retrospective. Single centre. Modest discrimination. Data-derived cut-off. Restricted to necrotising AP
Mofidi et al. [31] (IPN analysis)	2009	Systematic review and meta-analysis of prospective studies; 7 studies in the IPN analysis; study quality scored with STARD.	329 patients (see Table 2 for the SAP analysis)	Serial in the pooled studies: daily repeated measurement (3–14 days), highest value used Assays: BRAHMS immunoluminometric, RIA, PCT-Q strip	Study-level 0.48–3.5 (no single threshold)	80 (95% CI 70–87)	91 (95% CI 87–94)	Development of IPN; reference standard: positive FNA culture or intra-operative findings Pooled AUC 0.91; DOR 28.3; no significant heterogeneity.	Not tabulated at primary-study level	Not applicable at review level. Quality scored independently by the reviewers (STARD; κ = 0.79)	Secondary evidence. Includes the Rau et al. cohort. Studies pre-date 2009. Mixed assays and cut-offs

Abbreviations: * Cut-off derived from ROC, Youden or Phi-statistic optimisation in the same cohort (optimistic; not externally validated; Sens, sensitivity; Spec, specificity; AP, acute pancreatitis; AUC, area under the curve; BUN, blood urea nitrogen; CI, confidence interval; CRP, C-reactive protein; CT, computed tomography; DOR, diagnostic odds ratio; FNA, fine-needle aspiration; HCT, hematocrit; IPN, infected pancreatic necrosis; PCT, procalcitonin; RIA, radioimmunoassay; PCT-Q strip, Procalcitonin Quantitative test; SAP, severe acute pancreatitis; SIRS, systemic inflammatory response syndrome; STARD, Standards for Reporting Diagnostic Accuracy Studies; Sens, sensitivity; Spec, specificity; κ, Cohen’s kappa.

**Table 2:** Prognostic performance of PCT (target: severity, organ failure, mortality or composite infectious complications).

Study	Year	Design and Setting	n (Events)	PCT Sampling (Single vs. Serial)	Cut-Off (ng/mL)	Sens (%)	Spec (%)	Outcome Definition/Reference Standard	Blinding	Independent Adjudication	Principal Limitations
Kylanpaa-Back et al. [33]	2001	Prospective observational, single centre (Helsinki); consecutive patients with symptoms < 72 h	162 SAP = 38; organ failure = 22; deaths = 7	Serial, two time points: admission and 24 h (a 48 h sample was also drawn); results given for day 0, day 1 and the higher of the two PCT-Q semi-quantitative strip	>0.5 (strip band visible > 0.5; scored 0–3)	Day 1: 92 Day 0: 71	Day 1: 84 Day 0: 84	SAP vs. mild AP (Atlanta 1992 clinical classification); not IPN Day 1: NPV 97%, PPV 53%	Index test: Yes (strips read blind to clinical course by two trained technicians). Outcome assessor: Not reported.	Not reported.	Endpoint is SAP, not IPN. Semi-quantitative index test. 1992 Atlanta definitions (since revised)
Mofidi et al. [31] (SAP analysis)	2009	Systematic review and meta-analysis of prospective studies; 12 studies in the SAP analysis	826 (1001 unique patients across SAP and IPN analyses; 152 in both)	Mostly admission, 24 h or 48 h sampling; some studies daily up to 14 days Mixed assays	Study-level 0.25–1.8	72 (95% CI 64–77)	86 (95% CI 83–89)	SAP (Atlanta: necrosis or organ dysfunction) Pooled AUC 0.87; DOR 14.9; significant heterogeneity (Q = 28.56, *p* < 0.01)	Not tabulated at primary-study level	Not applicable at review level (κ = 0.79 for STARD scoring)	Substantial heterogeneity in timing, assay and cut-off. Overlaps with Kylänpää-Bäck et al. and Rau et al.
Rau et al. [14] (same cohort as Table 1)	2007	As Table 1	104 Infected necrosis + MODS = 28; deaths = 8 (7.7%)	Serial (as Table 1); early analysis on day 3 or 4	Day 3–4 ≥ 3.8 * Overall ≥ 3.5 *	Day 3–4: 79 (comp.); 86 (death) Overall: 93 (comp.)	Day 3–4: 93; 89 Overall: 88	Composite: infected necrosis + MODS or death (day 3–4: PPV 65%, NPV 97%); death alone MODS ≥ 2 failing organ systems needing ICU support	Not reported	Not reported	Composite endpoint mixes infection and mortality. Same limitations as Table 1.
Alberti et al. [29]	2022	Prospective single-centre cohort (Barcelona); 85.5% biliary aetiology; 81 patients excluded	152 IPN = 13; acute cholangitis = 20	Single time point: one admission sample (first 72 h); electrochemiluminescence immunoassay. Authors acknowledge the absence of repeat sampling	>0.68 * (source reports mg/dL)	IPN 76.9 Cholangitis 75.0	IPN 68.4 Cholangitis 70.2	Primary aim: infections (cholangitis, extrapancreatic, IPN), antibiotic use, urgent ERCP and severity. IPN = positive culture after necrosectomy or drainage, or clinical suspicion IPN: PPV = 18.9%, NPV = 96.9%	Not reported	Single time point. Only 13 IPN events (sensitivity 95% CI 49.7–91.8). IPN definition includes clinical suspicion. Data-derived cut-off.	

Abbreviations: * Data-derived cut-off (optimised in the same cohort). AP, acute pancreatitis; AUC, area under the receiver operating characteristic curve; CI, confidence interval; ERCP, endoscopic retrograde cholangiopancreatography; IPN, infected pancreatic necrosis; MODS, multiple organ dysfunction syndrome; NPV, negative predictive value; PCT, procalcitonin; PPV, positive predictive value; SAP, severe acute pancreatitis; STARD, Standards for Reporting Diagnostic Accuracy Studies; Sens, sensitivity; Spec, specificity; κ, Cohen’s kappa.

### 3.5. Comparison with Other Biomarkers

As summarized in Table 3, PCT has been investigated alongside conventional inflammatory biomarkers such as CRP, white blood cells (WBC), IL-6, and presepsin for differentiating IPN from sterile inflammatory responses. Available comparative evidence suggests that PCT may provide useful discriminatory information in selected clinical settings; however, direct comparisons between biomarkers remain heterogeneous because of differences in patient populations, timing of measurement, disease severity, assays, diagnostic thresholds, and reference standards [11,14,31,38].

CRP is constrained by delayed kinetics, generally reaching peak concentrations 48–72 h after symptom onset [4,17,25,39]. Although CRP is widely used as a marker of inflammatory severity, its ability to distinguish sterile inflammation from infection is limited [14,28]. Some studies in SAP have reported greater discriminatory performance of PCT than CRP for selected infection-related outcomes, although the relative sensitivity and specificity have varied according to the outcome, timing of sampling, and threshold used [14,31]. These findings should therefore not be interpreted as evidence of consistent superiority of PCT over CRP across all clinical settings [11,14,31]. Leukocytosis similarly lacks specificity because both sterile and infected necrosis can produce substantial inflammatory responses [32]. Thus, WBC counts should be interpreted in conjunction with other clinical, biochemical, and imaging findings rather than used alone to guide antibiotic decisions [40].

IL-6 provides useful information for early inflammatory and severity assessment and may rise rapidly during the initial phase of AP [11,18]. However, its short half-life may limit its usefulness for continuous monitoring, and routine clinical application may be constrained by assay availability [18,23]. Comparative studies of PCT and IL-6 have reported heterogeneous findings, partly because of differences in study populations, sampling time, and clinical outcomes [34,41]. Some studies have demonstrated useful discriminatory performance of PCT for infection-related outcomes, whereas IL-6 may provide valuable early information regarding inflammatory severity [34,41]. Accordingly, PCT should be considered a complementary biomarker rather than a uniformly superior alternative to IL-6.

Presepsin has also been investigated as an early biomarker of bacterial infection. In a prospective study of patients with severe acute necrotizing pancreatitis, presepsin concentrations were higher in patients with infected complications than in those with sterile necrosis, suggesting potential diagnostic utility for identifying infection [42]. However, this evidence is based on a relatively small single-center study, and the evidence base for presepsin in AP remains more limited than that for PCT. Therefore, current evidence does not support a consistent diagnostic advantage of presepsin over PCT in AP.

Overall, PCT may provide complementary information when bacterial infection is suspected, but its comparative diagnostic performance varies across studies and clinical settings. Differences in patient population, disease severity, timing of measurement, assay characteristics, diagnostic thresholds, and reference standards should be considered when interpreting comparisons with CRP, WBC, IL-6, and presepsin [11,14,31,38,42]. PCT is therefore best regarded as an adjunctive biomarker rather than a stand-alone or universally superior diagnostic test. Its dynamic behavior may additionally support serial assessment of disease progression and response to treatment [27,35].

**Table 3:** Comparative characteristics of biomarkers used in acute pancreatitis.

Biomarker	Primary Biological Trigger	Time to Rise After Stimulus (Hours)	Time to Peak	Biological Half-Life	Principal Clinical Role in AP	Specificity for Bacterial Infection	Major Limitations
PCT (Procalcitonin) [24,43]	Bacterial endotoxins and pro-inflammatory cytokines (TNF-α, IL-1β, IL-6)	2–4 h	24–48 h	25–30 h (20–24 h depending on assay platform)	Differentiation of IPN from sterile inflammation; guidance of antibiotic stewardship	Moderate-to-high, depending on clinical setting and threshold	May increase in severe systemic inflammation, major trauma, or renal dysfunction
CRP (C-Reactive Protein) [40]	Hepatic synthesis stimulated primarily by IL-6	6–12 h	48–72 h	~19 h	Assessment of disease severity and inflammatory burden	Low	Delayed peak limits early diagnosis; elevated in sterile inflammation
IL-6 (Interleukin-6) [44]	Tissue injury and cytokine activation (TNF-α, IL-1β)	<24 (typically 1–2 h)	2–24 h	<6 h (typically 1–4 h)	Early prediction of disease severity	Low	Short half-life; poor specificity for infection; limited routine availability
WBC (White Blood Cells) [45]	SIRS	Variable (typically 4–24 h)	Variable	Variable (circulating mature neutrophil half-life ~6–7 h before marginating)	General inflammatory assessment	Low	Markedly elevated in both sterile and infected inflammation
Presepsin (sCD14-ST) [46]	Monocyte activation following pathogen recognition	~2 h	2–3 h	4–5 h	Early detection of sepsis	Moderate-to-high in sepsis; limited evidence in AP	Limited validation in AP populations compared with PCT

Abbreviations: PCT, procalcitonin; CRP, C-reactive protein; IL-6, Interleukin-6; WBC, White Blood Cells; SIRS, systemic inflammatory response syndrome; IPN, infected pancreatic necrosis; TNF-α, tumor necrosis factor-alpha; IL-1β, interleukin-1 beta; sCD14-ST, soluble CD14 subtype (presepsin).

### 3.6. Antibiotic Stewardship

The excessive use of broad-spectrum antibiotics in AP is an ongoing global issue. Studies show that antibiotics are still widely used at rates of 31–80% even though the guidelines clearly state that routine prophylaxis should be avoided. Thus, this practice results in the development of microbial resistance, increased healthcare costs and the occurrence of undesired effects such as *Clostridioides difficile* infection and fungal complications [6,47]. One big clinical difficulty nowadays is that severe sterile SIRS generally cannot be visually or otherwise differentiated from sepsis, complicating antibiotic decision-making [29].

**Figure 3:**
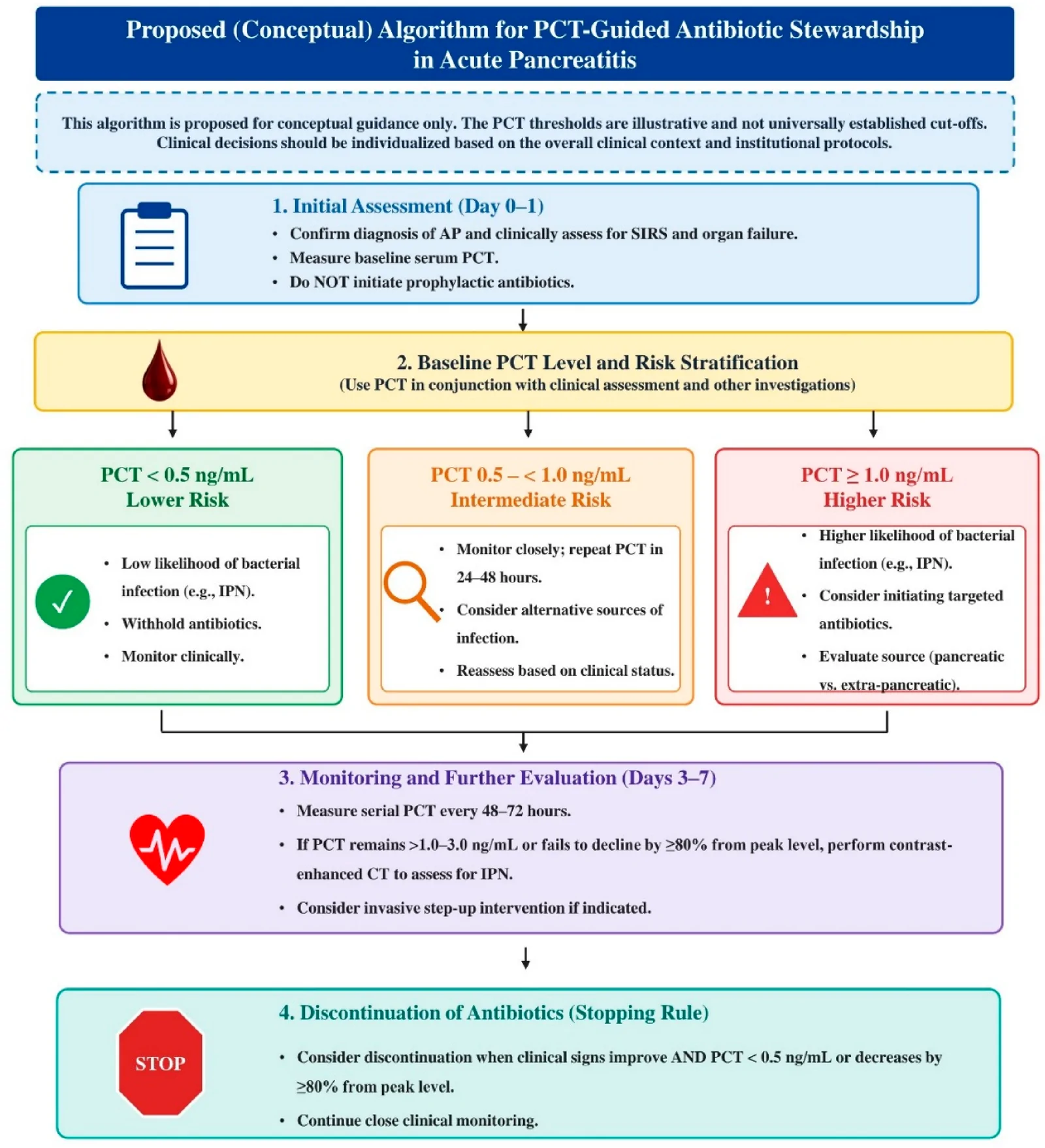
Context-dependent framework for PCT-guided antibiotic stewardship in acute pancreatitis. Abbreviations: PCT, procalcitonin; AP, acute pancreatitis; SIRS, systemic inflammatory response syndrome; IPN, infected pancreatic necrosis; CT, computed tomography; clinical decisions are guided by serum PCT thresholds, where levels < 0.5 ng/mL suggests low likelihood of bacterial infection, 0.5–<1.0 ng/mL indicate intermediate risk requiring monitoring, and ≥1.0 ng/mL indicate high likelihood warranting antibiotic initiation and further evaluation; persistent elevation or inadequate decline of PCT supports imaging and consideration of invasive intervention, while declining levels guide safe discontinuation of antibiotics.

Therefore, Antibiotic Stewardship Programs (ASPs) are turning more to PCT-guided algorithms to support decisions on whether or not to start or stop antimicrobial treatment. PCT-guided antibiotic algorithms have been investigated as a strategy to reduce unnecessary antibiotic exposure using predefined serum PCT thresholds. The PROCAP study was initially described in a 2019 study protocol, which evaluated a PCT threshold of 1.0 ng/mL for guiding antibiotic initiation and discontinuation [30]. The subsequent final PROCAP randomized controlled trial evaluated this algorithm in 260 patients with AP and compared PCT-guided care with usual care [48]. Other PCT-guided approaches, such as the study by Qu et al., used a lower threshold, with antibiotics initiated when overt clinical signs of infection were present and PCT concurrently exceeded 0.5 ng/mL [36] (Figure 3). Similarly, consensus statements from the International Association of Pancreatology (IAP) recognize that elevated PCT may provide supportive information when considering antimicrobial treatment, particularly when microbiological confirmation is unavailable [40]. The characteristics and outcomes of the available PCT-guided randomized trials are summarized in Table 4.

Stopping rules designed for the safe discontinuation of antibiotics may incorporate serial PCT measurements together with clinical assessment. In the PROCAP protocol, antibiotic discontinuation was planned when repeat PCT measurements on days 4 and 7 fell below 1.0 ng/mL in the absence of ongoing clinical concern [30]. Qu et al. reported discontinuation of therapy when clinical signs improved and PCT decreased to <0.5 ng/mL for three consecutive days [36] (Figure 3). Sepsis algorithms commonly recommend discontinuing antibiotics when PCT decreases by ≥80% from peak levels or falls below 0.5 ng/mL [49]. In the final PROCAP randomized controlled trial, antibiotic use decreased from 63% with usual care to 45% with PCT-guided care (adjusted risk difference −15.6%; *p* = 0.0071) [48]. Importantly, there was no significant increase in clinical infection, hospital-acquired infection, or mortality with PCT-guided management [48]. Similarly, Qu et al. reported shorter antibiotic duration in the PCT-guided group (10.9 ± 2.9 vs. 16.1 ± 2.5 days; *p* < 0.001), alongside reduced Intensive Care Unit (ICU) and hospital stay, without increases in infection, mortality, or adverse outcomes [36,49]. Observational evidence suggests that adherence to PCT-guided algorithms may reduce unnecessary exposure to broad-spectrum antibiotics, including carbapenems and fluoroquinolones [10]. Overall, these findings suggest that PCT-guided strategies may support antibiotic stewardship in selected patients with AP, although differences in thresholds, treatment algorithms, patient populations, and study settings should be considered when interpreting the available evidence (Table 4).

**Table 4:** PCT-guided antibiotic-stewardship randomised trials.

Study	Year	Design and Setting	n (Arms)	PCT Sampling (Single vs. Serial)	Algorithm: Start/Stop Thresholds	Comparator	Antibiotic-Exposure and Efficacy Outcomes	Safety Outcomes	Blinding	Independent Adjudication	Principal Limitations
Qu et al. [36]	2012	Randomised prospective single-centre trial (Huizhou, China); SAP (Atlanta), onset < 24 h	71 PCT-guided = 35; control 36	Serial: daily semi-quantitative immunoassay for up to 28 days, in the PCT-guided group only	Start: clinical signs of infection and PCT > 0.5. Stop: clinical improvement and PCT < 0.5 for 3 days	Antibiotic prophylaxis for 14 days (longer if infection confirmed)	Antibiotic duration 10.89 ± 2.85 vs. 16.06 ± 2.48 days (*p* < 0.001). ICU stay 11.11 vs. 14.83 days. Hospital stay 16.66 vs. 23.81 days. Cost US$24,401 vs. 27,813	Mortality 20.0% vs. 22.2% (*p* = 0.988). MODS 68.6% vs. 69.4% (*p* = 0.873)	Not reported. PCT measured only in the study group, so open-label in practice. Randomisation method and allocation concealment Not reported.	Not reported.	Small sample, underpowered for safety. Semi-quantitative PCT. Comparator was routine 14-day prophylaxis, which current guidelines discourage, so the effect size may not transfer to current practice
Siriwardena et al. (PROCAP) [30,48]	2019 (protocol); 2022 (results)	Pragmatic randomised single-centre trial (Manchester Royal Infirmary); adults with clinical diagnosis of AP; randomisation stratified by admission route	260 (results) PCT-guided = 132; usual care = 128 (protocol target 200)	Serial: day 0, then re-assay on days 4 and 7 for ward patients; daily in critical care; repeat when infection suspected. Fully automated Elecsys BRAHMS immunoassay	Antibiotics considered if PCT ≥ 1.0; withheld below 1.0 unless clinically justified. Cessation guided by PCT re-assayed at ≥48 h (48, 72 or 96 h) with no clinical evidence of infection	Usual care	Protocol primary: antibiotic use during the index admission (binary). Results: mean days of antibiotic use 5.8 vs. 4.5 (adjusted difference −1.16, 95% CI −2.10 to −0.22; *p* = 0.015)	Protocol secondary: all-cause mortality (non-inferiority), infections, multiresistant isolates. Deaths from severe pancreatitis 3% vs. 2%	Single-blind: patients blinded; treating clinicians not blinded. Outcome-assessor blinding not reported.	Not reported. An independent Data Monitoring Committee oversaw safety (not outcome adjudication)	Clinicians unblinded for a clinician-driven outcome (performance bias). Single centre. Clinician override permitted. Applicability to centres with different baseline antibiotic use is unknown

Abbreviations: AP, acute pancreatitis; CI, confidence interval; ICU, intensive care unit; MODS, multiple organ dysfunction syndrome; PCT, procalcitonin; PROCAP, Procalcitonin-guided Antibiotic Treatment in Acute Pancreatitis; SD, standard deviation. Values are presented as mean ± SD unless otherwise indicated.

## 4. Discussion

### 4.1. Critical Appraisal of Evidence

The diagnostic and prognostic value of PCT in AP is supported by a body of observational studies and meta-analytic evidence, although large definitive RCT data remain limited. Meta-analyses demonstrate moderate-to-high diagnostic accuracy in some settings for predicting IPN and SAP, with pooled analyses suggesting that PCT may provide useful diagnostic information and may offer earlier indication of infectious complications than CRP in selected populations [11,33,39]. However, these findings should not be interpreted as evidence of consistent superiority over CRP or other biomarkers, as direct head-to-head comparisons remain limited and heterogeneous with respect to patient populations, timing of measurement, assay characteristics, thresholds, and clinical endpoints. Importantly, the final PROCAP randomized controlled trial provided clinical evidence that PCT-guided management reduced antibiotic prescribing from 63% with usual care to 45% with PCT-guided care, without a significant increase in clinical infection, hospital-acquired infection, or mortality [48]. However, the trial was single-centre, and further multicentre studies are needed to establish the generalizability of these findings across different healthcare settings.

### 4.2. Clinical Guidelines

The role of PCT in AP guidelines has evolved from an experimental biomarker to a potentially useful adjunct in selected clinical situations, although adoption remains inconsistent. The 2013 IAP/American Pancreatic Association (APA) guidelines describe PCT as a promising marker for pancreatic infection, particularly with persistent organ failure, while emphasizing that it should not be used as a standalone diagnostic test [8]. The 2019 World Society of Emergency Surgery (WSES) guidelines identify serum PCT as potentially valuable for predicting the risk of IPN and assign a Grade 1B recommendation to its use [5]. They emphasize that PCT may assist in distinguishing infectious from non-infectious causes of persistent systemic inflammation [5].

Additionally, recent international consensus statements aimed directly at reducing systemic antibiotic overuse suggest that elevated PCT levels during the early phase of AP may provide supportive information when considering antibiotic treatment, although antimicrobial decisions should remain integrated with clinical assessment and other diagnostic findings [40]. This is particularly important because elevated PCT alone does not establish microbiologically confirmed infection.

Despite these recommendations, significant gaps between evidence and daily clinical practice remain. Widespread implementation is hindered by variation in proposed PCT thresholds and the lack of universally accepted, actionable cut-off values within the guidelines themselves. While modern guidelines strongly discourage routine prophylactic antibiotics [2,5] they do not uniformly recommend PCT-guided algorithms as the standard of care, partly because of differences in assay availability, clinical protocols, healthcare resources, and cost compared with CRP testing [26]. Several recent reviews note that while PCT testing may have a role in supporting antimicrobial stewardship programs (ASPs), its effects in countries with already restrictive antibiotic policies remain to be fully evaluated [50].

### 4.3. Limitations of Procalcitonin

While PCT may provide useful diagnostic information for suspected bacterial infection in selected clinical settings, its interpretation is subject to several physiological and analytical limitations. Recent evidence indicates that the diagnostic performance of PCT varies according to the timing of measurement and clinical context. In a systematic review and meta-analysis of acute necrotizing pancreatitis, PCT and CRP demonstrated similarly limited predictive value within the first 72 h after admission, whereas both biomarkers showed greater predictive value after 72 h, with substantial variation in pooled estimates [11]. Comparative findings should therefore be interpreted cautiously because direct head-to-head comparisons between PCT and other biomarkers remain heterogeneous and may be influenced by differences in patient populations, disease severity, timing of measurement, assay characteristics, diagnostic thresholds, and reference standards [11]. Accordingly, PCT should not be interpreted as a universally superior biomarker, and its diagnostic value is best considered in conjunction with clinical assessment, imaging findings, and other laboratory markers. PCT levels can be falsely elevated in the absence of confirmed bacterial infection, particularly during severe systemic inflammation. During the initial days of SAP, marked inflammatory responses associated with SIRS may contribute to PCT elevation even without documented infection [7]. Markedly high PCT levels may also be observed following severe blunt trauma, major open surgery, severe burns, and prolonged cardiogenic shock [23,26]. Renal dysfunction is an additional important consideration because acute kidney injury (AKI) is common in SAP and may be associated with increased circulating PCT concentrations. Impaired renal function may alter PCT concentrations through reduced clearance and other mechanisms, potentially affecting the interpretation of conventional PCT thresholds [24,51]. Therefore, PCT values should be interpreted in conjunction with renal function and the overall clinical context, particularly in patients with AKI or significant renal impairment.

Diagnostic blind spots also exist. PCT may remain falsely low in highly localized infections that fail to elicit a substantial systemic inflammatory response [24,52]. A deeply seated infected necrotic collection might not immediately trigger a systemic PCT rise if infection is localized or contained, or if it is caused by organisms associated with a weaker PCT response [26,27].

The lack of high-quality evidence for the interpretation of PCT is further compounded by methodological issues in studies found in the literature. A Cochrane review on the subject reported that only a few high-quality studies were available to evaluate the diagnostic accuracy of PCT as a standalone triage test, including only three diagnostic studies with a total of 242 participants, resulting in wide confidence intervals for sensitivity (0.51–0.97) [53]. Such small study samples reduce the precision of diagnostic estimates and may limit the generalizability of findings to different pancreatitis populations and etiologies [54]. There is also a lack of assay standardization and variation in assay platforms, which may contribute to differences between studies and affect comparability. Earlier studies used semi-quantitative strip tests (e.g., PCT-Q), which provide results by visually interpreting color changes corresponding to broad PCT concentration ranges (<0.5, 0.5–2, 2–10, >10 ng/mL) [33]. While these kits are rapid, they provide less precise quantitative measurements than modern immunoassays, such as Kryptor or chemiluminescence-based platforms used in more recent studies [26,55].

Lastly, cost and laboratory availability represent additional practical considerations. Although conventional quantitative immunoassays may reduce some of the variability associated with semi-quantitative testing, differences between assay platforms can still affect measured concentrations and the applicability of thresholds across settings. Semi-quantitative strip tests offer rapid results but may provide less precise measurements than quantitative assays, which can be relevant when applying PCT thresholds to antimicrobial stewardship strategies [26,56]. In addition, PCT assays are generally more expensive than standard CRP testing, which may limit routine availability in resource-constrained settings [57].

### 4.4. Future Directions

To integrate PCT widely into the routine management of AP, several uncertainties regarding its clinical use and interpretation need to be addressed. The reported cut-off values show substantial variation, with thresholds ranging from >0.5 ng/mL for infection risk assessment to >3.8 ng/mL for predicting organ failure, emphasizing the need for standardized, clinically validated and risk-stratified thresholds for assessing infected necrosis, supporting antibiotic decisions, and discontinuing treatment [14,19,35].

Although the final PROCAP randomized controlled trial provided evidence supporting the potential role of PCT in antibiotic stewardship, further multicenter and multinational prospective studies are needed to determine the safety, effectiveness, and generalizability of PCT-guided strategies across different healthcare settings and to compare escalation and de-escalation approaches [48,50]. Future studies should also evaluate whether combining PCT with clinical markers or established scoring systems, such as APACHE II and the Bedside Index for Severity in Acute Pancreatitis (BISAP), improves predictive performance [58,59]. Such approaches may ultimately position PCT as one component of a multimodal prognostic or antimicrobial-stewardship strategy rather than as an isolated biomarker.

Emerging artificial intelligence-assisted and data-driven approaches may further enhance antimicrobial optimization by integrating clinical, microbiological, pharmacokinetic, and biomarker information to support individualized treatment decisions [60]. In AP, serial PCT measurements could potentially be incorporated into such multimodal decision-support models together with clinical trajectory, microbiological findings, organ function, and imaging to inform decisions regarding antibiotic initiation, continuation, de-escalation, and discontinuation. Such an approach represents a potential future application of data-driven antimicrobial stewardship in AP rather than an established clinical strategy. However, these models would require prospective development and validation specifically in AP populations before their routine clinical implementation.

## 5. Conclusions

PCT has emerged as a potentially valuable adjunctive biomarker in the management of AP, particularly for differentiating IPN from sterile inflammation. Compared with conventional inflammatory markers such as CRP and leukocyte count, PCT has generally shown greater diagnostic specificity for bacterial infection in relevant clinical settings and may provide clinically useful information during the early stages of disease. In addition to its diagnostic role, elevated or persistently increasing PCT concentrations have been associated with severe AP, organ failure, sepsis, and mortality, supporting its potential role in early risk stratification. The available evidence also suggests that PCT-guided antibiotic strategies may reduce unnecessary antibiotic exposure without adversely affecting clinical outcomes in selected settings, thereby contributing to antimicrobial stewardship. However, PCT should be interpreted in conjunction with clinical assessment, laboratory findings, and imaging studies rather than as a standalone diagnostic test. Variability in reported diagnostic thresholds, study populations, assays, and clinical protocols currently limits the universal application of PCT-guided decision-making. Overall, current evidence supports the use of PCT as an adjunct rather than a standalone tool in the diagnosis and management of AP. Future large-scale prospective multicenter studies are needed to establish standardized cut-off values, validate PCT-guided clinical algorithms, and further define its role in routine clinical practice.

## Data Availability

No new data were created or analyzed in this study. Data discussed in this review are available in the cited publications.
